# Classic Illusion Sheds New Light on the Neural Site of Tactile Perception

**DOI:** 10.1371/journal.pbio.0040096

**Published:** 2006-02-28

**Authors:** Liza Gross

Imagine a race of people with tiny torsos, arms, and legs, but gigantic fingers, lips, and tongues. That's what we would look like if each of our body parts were sized proportionally to the brain area that processes their sensory inputs. Each of our body parts is represented in the somatosensory cortex on a map (called the somatotopic map), which expands the representation of the more sensitive body parts. Sensory inputs travel from touch receptors in the skin to neurons in the appropriate sectors of the somatosensory cortex.

As much as neuroscientists know about these neural projections for touch, surprisingly little is understood about the neural correlates of conscious tactile perception. In a new study, Felix Blankenburg, Jon Driver, and their colleagues turn to a classic somatosensory illusion—called the cutaneous rabbit—that is perfectly suited to decoupling real and illusory touch. In the illusion, a rapid succession of taps is delivered first to the wrist and then to the elbow, which creates the sensation of intervening taps hopping up the arm (hence the illusion's name), even when no physical stimulus is applied at intervening sites on the arm.

Blankenburg et al. took advantage of this somatosensory illusion to investigate which brain regions play a role in illusory tactile perceptions. Previous studies had implicated the somatosensory cortex in the rabbit illusion, but did not directly test this possibility. To do this, the authors used state-of-the-art functional magnetic resonance imaging technology (called 3T fMRI) to scan the brains of people experiencing the illusion. With the enhanced image quality and resolution of this scanner (deriving from the stronger magnetic field plus a specially customized imaging sequence), the authors show that the same brain sector is activated whether the tactile sensation is illusory or real.

To identify brain-related activity associated with real and illusory perceptions, the researchers taped three electrodes to the inner side of participants' left forearms, one just above the wrist, the others spaced equidistantly toward the elbow. Electrical stimulation could be applied to these points (P1, P2, and P3) while participants lay in the scanner. For the genuine rabbit experience, each point received three pulses in P1-P2-P3 succession. For the illusion, six pulses were applied to P1 (the last three substituting for pulses at the intervening P2 site), followed by three pulses to P3; this resulted in the same P1-P2-P3 tactile experience as when P2 was actually stimulated. For the control condition, P1 received three pulses, followed by three pulses to P3 and then to P1 (a sequence that does not produce the illusion of being stimulated at P2). After each sequence, participants indicated whether or not they felt any stimulation at P2.

Blankenburg et al. looked for brain regions that showed similar increases in neuronal activity during the real and illusory rabbit conditions, compared with the controls, and also looked for any regions that differed between the two conditions. Only one area showed similar and heightened activity during the genuine and illusory rabbit sequences, compared with controls: the precentral gyrus, where the first cortical area to represent touch is located (called S1). The increased activity within S1 fell in the exact sector corresponding to the P2 position on the forearm (even though it was not actually stimulated during the illusion). The researchers confirmed this correspondence by separate somatotopic mapping of the skin sites' representation in each participant's brain when each site was stimulated (with no illusion produced).

Altogether, these results suggest that the illusion of being touched at a particular place on the body engages exactly the same sector of the brain that would respond if that body part had actually been touched. This connection between conscious perception and somatotopic cortical processing for illusory percepts may shed light on conditions such as phantom limb pain following amputation, and other perceptual illusions associated with disease. The authors point out that recent fMRI studies have shown somewhat analogous effects in the visual system, with the primary visual cortex involved in some conscious visual illusions. It's still unclear if this phenomenon will hold for all other perceptual systems as well, but future studies can now explore how the brain bridges the gap between actual stimulation and conscious experience.

## 

**Figure:**
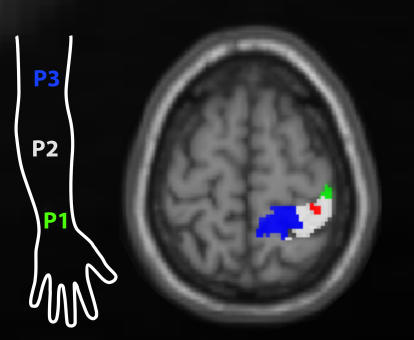
The cutaneous rabbit illusion engages the same sector of the brain that would respond if that body site (P2) had actually been touched.

